# Neck Neurofibromatosis: clinical and surgical features

**DOI:** 10.1016/S1808-8694(15)31079-X

**Published:** 2015-10-22

**Authors:** Clarissa L. Buono Lehoczki, Ronny Tah Yen Ng, Reinaldo Jordão Gusmão

**Affiliations:** 1 SBORL Specialist 2004, otolaryngologist, MD at the Pediatric Otolaryngology Department at Unicamp. Mailing address: Clarissa Buono Lehoczki - Al. Arapanés 113 Moema São Paulo SP 04524-000.; 2 Resident of Otolaryngology at Unicamp.; 3 PhD in Otoralyngology at FCM UNICAMP, Head of the Pediatric Otolaryngology Department at UNICAMP. Universidade Estadual de Campinas.

**Keywords:** neck, neurofibromatosis, treatment

## INTRODUCTION

Type I Neurofibromatosis (NF1) is a dominant autosomal disease. Its prognosis is related to the development of tumors that may evolve to malignancy. It is characterized by multiple café-au-lait spots, skeletal defects, optical gliomas, Lisch nodules and neurofibromas.^1^

Plexiform neurofibromas (PN) are one of the many possible findings on patients affected by the disease.

This paper aims at reporting on a case of a pediatric patient with cervical plexiform neurofibromas and the difficulties inherent to the treatment.

## CASE STUDY

A four months old male patient, with high respiratory discomfort, was diagnosed with a mass in the rhinopharynx that extended all the way to the oropharynx, later described as a plexiform neurofibroma in the pathologist's report.

The CT scan of the area revealed a mass located in the left parotid region. The tumor was diverting the internal carotid artery from its original position and compressing the internal jugular vein. (Picture 1)

At 16 months of age the patient underwent a tracheotomy to address the respiratory difficulties. At 18 months he was submitted to a partial excision of the tumor as it was infiltrating in the cervical plexus, brachial plexus, and facial nerve. Biopsy findings also pointed to plexiform neurofibroma.

The child is being monitored for worsening of compressive symptoms and signs of malignancy.

## DISCUSSION

Patients with NF1 have increased chance of developing benign and malignant tumors, which may in some cases infiltrate and lead to compression of vital structures and evident deformities.^2^

Images are key for diagnostic purposes, and tissue biopsy is used to confirm the diagnosis.

Although most tumors are benign,

**Figure:**
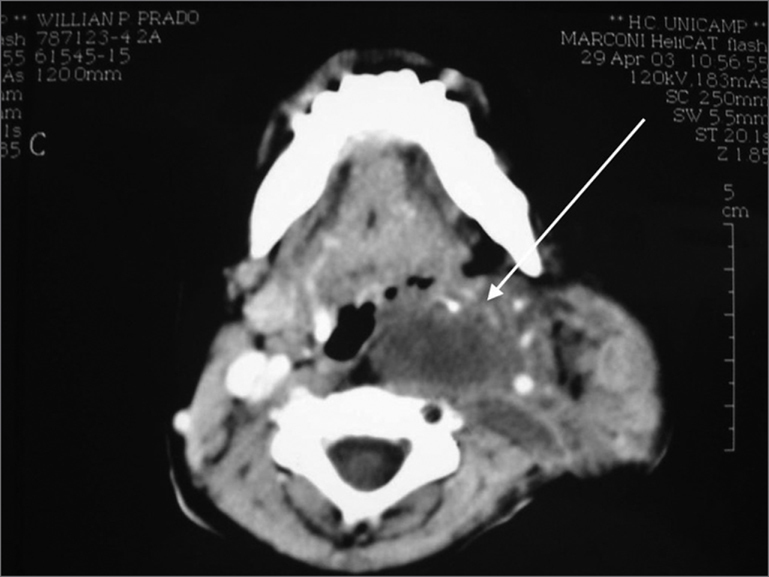
Cervical Neurofibromatosis - see arrow for tumor in neck CT scan axial view.

the risk of malignancy ranges between 4 and 12% as described in the literature.^3^

Surgery is the only effective option to manage plexiform neurofibromas. Success is however limited, as this is a highly infiltrating tumor with elevated relapse rates. Besides, the difficulty in identifying the main nervous plexus during surgery increases postoperative morbidity and incidence of neurological complications.^4^

It seems prudent to postpone surgery in symptom-free patients with head and neck PN until clearly obstructive symptoms are present.^5^

## CONCLUSIONS

Neurofibromatosis is an entity to be considered by otolaryngologists when performing the differential diagnosis of cervical tumors.

It usually manifests itself in the form of deeply located benign tumors that may compress vital structures. In such cases, surgical management is required.^6^

There is consensus that surgery must be postponed until compressive symptoms are present or signs of malignancy have been identified.
